# Oral mucosal lesions in teenagers: a cross-sectional study

**DOI:** 10.1186/s13052-017-0367-7

**Published:** 2017-05-31

**Authors:** Francesca Amadori, Elena Bardellini, Giulio Conti, Alessandra Majorana

**Affiliations:** 1 Dental School, Pediatric Dentistry Department, University of Brescia, P.le Spedali Civili, 1, 25123 Brescia, Italy; 2 University Vita-Salute San Raffaele, Milan, Italy; 3 Clinica Odontoiatrica, P.le Spedali Civili 1, 25123 Brescia, Italy

**Keywords:** Epidemiology, Oral mucosal lesion, Adolescent

## Abstract

**Background:**

Adolescence is a period of transition to adulthood. Little is known about oral mucosal lesions (OMLs) in teenagers, in which the emergence of new habits, unfamiliar to children, could affect the type of lesions.

The aim of this study was to evaluate the distribution of oral mucosal lesions (OMLs) in a wide sample of adolescents.

**Methods:**

A retrospective cross-sectional study was carried out examining all medical records of adolescents (aged 13–18 years) treated at the Dental Clinic of the University of Brescia (Italy) in the period from 2008 to 2014. Cases with OMLs were selected. Data regarding age, gender, type of OML, bad habits, systemic chronic diseases were collected.

**Results:**

A total of 6.374 medical records (mean age 15.2 + −1.7 years) were examined. We found 1544 cases (31.7%) of oral mucosal lesions; 36 different types of mucosal alterations were detected and the most frequent were: aphthous ulcers (18%), traumatic ulcerations (14.3%), herpes simplex virus (11%), geographic tongue (9.6%), candidiasis (5.5%), and morsicatio buccarum (4.7%). Papilloma virus lesions (1.7%), piercing-related lesions (4%), multiform erythema (0.13%), oral lichen planus (0.13%) and granular cell tumour (0.06%) were also diagnosed.

**Conclusions:**

The prevalence of OMLs in adolescents are different from those in children and, in some conditions, it could increase with age.

## Background

In literature, epidemiological studies of oral mucosal lesions (OMLs) are still poor, if compared with reports regarding dental caries or periodontal diseases [1]. This gap is even more apparent in case of children and adolescents, where studies focus above all on cancer patients or on samples with specific chronic diseases [2, 3]. In addition, there is a tendency of using different experimental methods, non-standardized diagnostic criteria and small samples which leads to a controversial and underestimated prevalence of OMLs in adolescents [4, 5]. However, literature demonstrates that OMLs prevalence seems to change and increase with age along with the development of bad habits [1, 6].

The investigation of OMLs prevalence in specific population groups is mandatory in order to understand its extension and characteristics, but it is also important for the improvement of oral health promotion and prevention programs for specific age groups, as recommended by the World Health Organization [7].

In a previous report [2], we evaluated the prevalence of OMLs in a wide sample of children, including both those who were healthy and those who had a chronic disease, aged 0–12 years. This study focuses on a different age group, which is that of teenagers, in order to determine the prevalence of OMLs in adolescence.

## Methods

### Study population

This study was designed as a retrospective cross-sectional study. All the medical records of adolescents (aged 13–18 years) who were treated at the Dental Clinic of the University of Brescia (Italy) in the period from January 2008 to December 2014 were reviewed in order to select teenagers with OMLs. The medical records of patients with lesions linked to dental caries, periodontal diseases and endodontic problems were excluded from the study.

### Medical records forms

The medical records of the patients with OMLs were filled out by three clinicians who had undergone the same training and therefore permitted the standardization of the procedures. The calibration to detect OMLs was the same as our team’s previous study [2]; each examiner repeated the calibration every 2 years (three times during the study period). Each patient’s examination was conducted on a dental chair, under artificial lighting and with a mirror. Oral mucosal lesions were classified following the WHO criteria [8]. When necessary, complementary laboratory tests were performed.

### Data collection

The following data were recorded from each patient’s medical records: age at time of diagnosis, gender, smoker or non-smoker (if smoker, were they a current or former one), alcohol consumption, systemic chronic diseases, use of drugs and the clinical aspects of the OMLs (classification, location, symptoms, medications). The clinical data of the adolescents were recorded on a specifically designed chart.

### Data analysis

The data were inserted on a spread sheet. A 5% level of significance was used and the data was analysed using R® software for Mac. Descriptive analysis, bivariate analysis and Fisher’s test were used.

### Ethical considerations

Parents or caregivers signed an informed consent before the beginning of the study. Helsinki Declaration guidelines were followed for this analysis.

## Results

A total of 6.374 medical records belonging to adolescents (mean age 15.2 + −1.7 years) were examined (3387 boys and 2987 girls). Out of the total sample, 1544 (31.7%) teenagers showed OMLs. Sociodemographic data are shown in Table 1.

**Table 1:** Demographic and behavioral features of the patients with OMLs (*n* = 1544)

	Number	Percent
Gender		
Male	587	38.1
Female	957	61.9
Smoking		
Smoker	332	21.5
Non-smoker	1212	78.5
Alcohol (3 or more glasses/week)		
Yes	803	52
No	741	48
Systemic diseases		
No	939	60.8
Yes	605	39.2

Mean age 14.7 ± 0.8 years

Out of the 1544 adolescents, 36 types of OMLs were detected and the most frequent were: aphthous ulcers (*n* = 278; 18%), traumatic ulcerations (*n* = 221, 14.3%), herpes simplex virus (*n* = 170, 11%), geographic tongue (*n* = 148, 9.6%), candidiasis (*n* = 85, 5.5%), and morsicatio buccarum (*n* = 73, 4.7%). Papilloma virus lesions (1.7%), piercing-related lesions (4%), multiform erythema (0.13%), oral lichen planus (0.13%) and granular cell tumour (0.06%) were also diagnosed.

39.2% of the patients (*n* = 605) affected by OMLs also suffered from some form of systemic disease. The distribution of OMLs in healthy patients and in adolescents with systemic diseases is shown in Table 2.

**Table 2:** OMLs in healthy patients and in adolescents with systemic diseases

Type of oral lesion	Tot (%)	Healthy	Systemic diseases	OR (95% CI)	*p*-value
Apthous ulcers	278 (18%)	122	156	2.33 (1.79–3.03)	<0.001*
Traumatic ulcers	221 (14.3%)	113	108	1.59 (1.19–2.11)	0.0014*
Herpes Simplex Virus infection (HSV)	170 (11%)	93	77	1.33 (0.96–1.83)	0.0836
Geographic tongue	148 (9.6%)	98	50	0.01 (0.01–0.02)	<0.001*
Candidiasis	85 (5.5%)	31	54	2.87 (1.82–4.52)	<0.001*
Morsicatio buccarum	73 (4.7%)	71	2	0.04 (0.01–0.17)	<0.001*
Fordyce’s granules	66 (4.3%)	37	29	0.98 (0.6–1.61)	0.9401
Focal Hyperkeratosis	65 (4.2%)	49	16	0.49 (0.29–0.88)	0.0140*
Piercing lesions	62 (4%)	62	0	-	<0.001*
Mucocele	51 (3.3%)	48	3	0.09 (0.03–0.3)	<0.001*
Peripheral fibroma	43 (2.8%)	34	9	0.4 (0.19–0.84)	0.0129*
Gingival hyperplasia	36 (2.3%)	0	36	-	<0.001*
Physiologic pigmentation (ethnic)	34 (2.2%)	34	0	-	<0.001*
Hairy tongue	32 (2.1%)	20	12	0.93 (0.45–1.92)	0.8432
Linea alba	30 (1.9%)	27	3	1.59 (1.19–2.11)	0.0014*
Congenital hemangiomas	27 (1.75%)	22	5	0.35 (0.13–0.92)	0.0265*
Oral wart	26 (1.7%)	8	18	3.57 (1.54–8.26)	0.0015*
Actinic cheilitis	23 (1.5%)	8	15	2.96 (1.25–7.02)	0.0100*
Pyogenic granuloma	21 (1.4%)	21	0	-	<0.001*
Smoking-associated melanosis	11 (0.7%)	11	0	-	0.0075*
Drug reactions	7 (0.45%)	5	2	0.62 (0.12–3.29)	0.5643
Leukoedema	6 (0.4%)	6	0	-	0.0488*
Iron deficiency anemia	5 (0.3%)	0	5	-	0.0053*
Contact allergies	4 (0.25%)	4	0	-	0.018*
Petechiae and ecchymoses	3 (0.2%)	0	3	-	0.0308*
Heavy metal pigmentation	3 (0.2%)	3	0	-	0.164
Amalgam tattoo	2 (0.13%)	2	0	-	0.256
Erythema multiforme	2 (0.13%)	2	0	-	0.256
Oral lichen planus	2 (0.13%)	2	0	-	0.256
Pleomorphic adenoma	2 (0.13%)	2	0	-	0.256
Neurofibroma	1 (0.06%)	0	1	-	0.212
Necrotizing sialometaplasia	1 (0.06%)	1	0	-	0.422
Herpes Zoster infection	1 (0.06%)	1	0	-	0.422
Pemphigus vulgaris	1 (0.06%)	1	0	-	0.422
Dermatitis herpetiforme	1 (0.06%)	0	1	-	0.212
Granular cell tumour	1 (0.06%)	1	0	-	0.422

* = significant, p < 0.05

These systemic diseases included diabetes (10%), asthma (8%), heart disease (4%), organ transplant (5%), encephalopathies (9%), gastrointestinal diseases (16%), nephropathies (4%), syndromes (9%), blood diseases (21%) and primary and secondary immunodeficiencies (14%). In particular, of the gastrointestinal diseases, celiac disease was the most common. The adolescents with primary immunodeficiencies suffered from Di George syndrome, Hyper-IgE syndrome or IgA deficit T and B cell immunodeficiencies. Acquired immunodeficiencies were secondary to HIV infection. Teenagers with blood diseases had anemia, platelets deficit, leukaemia or lymphomas.

**Fig. 1:**
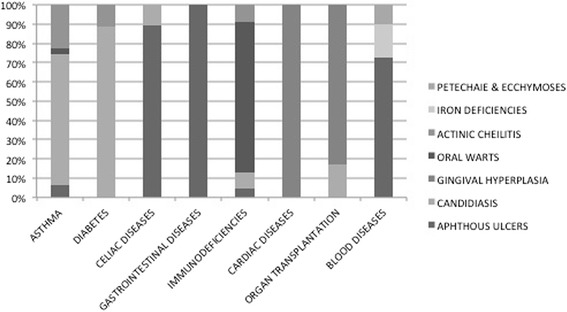
Association between OMLs and the main systemic diseases

The association between OMLs and the main systemic diseases is displayed in Fig. 1.

## Discussion

This retrospective study describes the distribution of OMLs among teenagers who were/are patients of the Dental Clinic of the University of Brescia over a 6-year period. Before discussing the findings of this study, it should be explained that the sample included adolescents admitted both as outpatients for dental emergencies and as inpatients for dental care or consultation from other departments of the Spedali Civili of Brescia (i.e. general pediatrics, pediatric hematology and oncology, auxology, etc.…). The sample is therefore heterogeneous, including both healthy and diseased adolescents. The percentage of healthy people included in this study (61.8%) is almost double compared to a previous study carried out in the same clinic, which focused on children from 0 to 12 years old (39.3%) [2]. The reason lies in the greater number of outpatient teenagers compared to inpatient teenagers i.e. diseased patients from internal departments of the hospital. Most requests of internal consultation from the dental clinic are for the 0–12 age range. With increasing age, health problems tend to stabilize, thus requiring less hospital check-ups and so the diseased adolescents begin to be treated by private dentists.

Comparison with other reports is not easy, due to the different socio-demographic characteristics of the samples (e.g. schoolchildren or clinic attendees), dissimilar clinical diagnostic criteria and methods, i.e. examination settings and calibration among examiners [9]. These differences should be taken into consideration when comparing the frequency and distribution of OMLs in these two age groups (ages 0–12 versus 13–18). Our study reports a prevalence of OMLs of more then 31% in teenagers; this data is in accordance with literature: a report carried out in Turkey only on adolescents [5] found a slightly lower prevalence (26.2%) and our previous study [2] reported a prevalence of 28.9% in a younger sample (0–12 years). Instead, a study done in the USA [10] showed a prevalence of 10.26% of OMLs, but in a wider age range sample (2–17 years old children). The prevalence rate of OMLs increases with age [1, 11] is most likely due to the deterioration of health status and associated to certain behaviors such as smoking and/or drinking. In our experience, we found the prevalence in the two age ranges of 0–12 years and 13–18 years to be quite similar, but it could be due to the fact that the percentage of chronic diseases potentially associated to oral lesions was higher in the previous and younger study group. The association between OMLs and systemic disease in the adolescent patients is reported in Table 2.

In literature, the most commonly reported OMLs are geographic tongue, herpetic stomatitis and oral ulcers [9]. These lesions were in fact the most frequent among the adolescents of this study (Tab. 2).

The most common type of OML observed in the teenagers was recurrent aphthous stomatitis (RAS) (18%). The general prevalence of this lesion has a wide range varying from 0.5% to 39.2%, but it reduces to 10.8% in studies recording their findings on the same day the patient was examined [9]. Since the ulcers may occur at intervals and usually heal within 2 weeks [12], its lifetime prevalence is frequently underestimated [13]. In accordance with literature [14], in this report RAS resulted significantly frequent (*p* < 0.005) in teenagers affected by systemic diseases, in particular those with gastrointestinal disturbances (i.e. coeliac disease) and anemia. RAS was more frequent in girls when compared to boys (*p* < 0.005). This result is in contrast with the findings reported by Parlak et al. [5] who found no significant differences between the two sexes.

Traumatic lesions were the second most frequently detected (14.3%). The prevalence of lesions caused by trauma found in this study is difficult to compare, because not every study investigated this type of alteration [5] or they would include it in a wider group of lesions [1, 9]. Traumatic ulcers result from heterogeneous causes i.e. from physical, thermal or chemical injuries. Accidental biting during mastication or rough-edged/hot food may cause acute traumatic ulceration, as well as overzealous tooth brushing or iatrogenic damage caused by dental treatment. Such ulcers generally heal within a few days with no complications. However, chronic trauma caused by sharp edges on teeth, restorations and orthodontic appliances may cause chronic ulcers. The majority of such injuries were unintentional. We found some cases of self-inflicted injuries, i.e. in 2 cases of epilepsy attack [15].

Chronic biting (nibbling) of the buccal mucosa often leads to loose threads such as keratin shreds, tissue tags or desquamative areas on the mucosal surface [16]. Such lesions have been referred to as “morsicatio buccarum” when it occurs on the buccal mucosa, “morsicatio labiorum” when on the labial mucosa and “morsicatio linguarum” when it is on the lateral borders of the tongue [17]. A significant percentage of adolescents in our group had this type of reactive OML, which was usually linked to a period of stress and intense studying. We found no differences regarding gender or state of health.

The infection of Herpes Simplex Virus (HSV) was found in 11% of the sample. Even if this result is slightly in contrast to literature [18], it highlights the fact that it is difficult to calculate the exact prevalence because of the characteristic intermittence of the lesions and the different precipitating factors (sun exposure, antibiotic treatments etc.…). Another confounding factor is the methodology used to diagnosis this viral infection: through clinical observations, serological assay or using saliva samples [19, 20]. Despite immunosuppression being one of the causes of HSV reactivation, no difference was recorded between healthy adolescents and those with chronic diseases (*p* = 0.08) in our sample. The same was for smoking, which in our sample did not seem to be a precipitating cause.

Geographic tongue, also called benign migratory glossitis, was found to be more frequent in adolescents when compared to previous reports in children [2, 5]. In our previous study on children under the age of 13 there seemed to be an association between geographic tongue and chronic diseases [2], while in the present sample there is a significant link with healthy adolescents. These finding seem to support the still unclear etiology of the pathology.

About 15.7% of teenagers (*n* = 243/1544) of the sample were smokers. In Italy, as in many other parts of the world, tobacco use by young people remains a serious problem: by the age of 15 years, over 50% have already tried smoking and nearly 15% are daily smokers. Furthermore, the global youth tobacco survey’s (GYTS) data on susceptibility to start smoking within 1 year, among those who have not yet started, indicate that 35.4% of the males and 46.6% of the females fall into this susceptible category [21]. We found that smoking in teenagers was significantly associated with hairy tongue, smoking-associated melanosis, and focal hyperkeratosis (*p* < 0.05%). This confirms that smoking has an effect on oral mucosa which may lead to different OMLs, by altering the microbial flora, with proliferation of fungi and chromogenic bacteria and over growth of the lingual papillae, or by stimulating the melanocytic pigment extravasation or by promoting the thickening of the oral mucosa.

It is interesting to point out that OMLs normally not described in children and adolescents were found in this sample; in particular, piercing related lesions (4%), oral lichen planus (0.13%) and granular cell tumour (0.06%).

Piercing related lesions consisted of abrasions or thickening of the oral mucosa in areas corresponding to points of contact with jewelry, usually on the interior side of the lips or on the surface of the tongue. A small percentage of the lesions was due to trauma caused by piercings no longer present. Literature focuses above all on gingival thickness and recessions [22] but, since there has been growing attention to piercing aesthetics in adolescents the last few years, we can speculate that further studies about OMLs will detect an increasing number of these lesions.

Granular cell tumour (GCT) often affects people aged 40–60, with a male female ratio of 1:2. It is rare in childhood, and usually affects individuals ages 10–18 [23]. Although most of GCTs are benign, a very small percentage can be malignant and can also metastasize [24], therefore its early detection is important, especially at such a young age.

In this analysis, we detected only one adolescent (0.06%) with oral lichen planus (OLP); literature estimates that OLP affects 1–16% of patients younger than the age of 15, particularly in cases of chronic disease, stress and infections [25]. In this sample, the patient did not have a chronic disease; however, both in healthy and in diseased patients, a prompt diagnosis and treatment are mandatory to prevent complications.

Some types of OMLs (Fig. 1) resulted significantly more frequent in the adolescents affected by systemic diseases, as a direct consequence of the pathology or as an indirect effect of the pharmacological therapy to cure it. As previously mentioned, aphthous ulcers resulted directly related to some diseases, in particular to celiac disease, to other intestinal diseases and to blood diseases (e.g. anemia). This association seems to confirm that- even if the cause of aphthous ulcers remains unknown- several etiologic factors, like nutritional intolerance or iron deficiencies, seem to play an important triggering role [26]. Oral candidiasis depended both on the diseases (i.e. immunodeficiencies and diabetes) and both on the pharmacological treatment, as in the case of asthma (due to inhaled or topical corticosteroids) and of organ transplantation (due to immunosuppressive drugs). This finding reflects the complex relationship between the commensal state and the pathogenicity of this organism (based on local factors in some cases and systemic factors in others), which can determine the transformation from a state of commensalism to pathogen [26]. Gingival hyperplasia, an overgrowth of gingival fibrous connective tissue, is the result of an unusual tissue response to chronic inflammation. This OML resulted predominantly linked to heart diseases and organ transplantation for the hyperplastic effect respectively of antihypertensive/antiarrhythmic drugs and of immunosuppressive drugs, often associated with local factors, such as bacterial plaque. Oral warts resulted more common among adolescents with immunodeficiencies. Some of oral squamous papillomas have been associated with the same human papilloma virus (HPV) subtypes that cause cutaneous warts. Other oral papillomas have been associated with different HPV subtypes [26]. Whether all oral papillomas are of viral aetiology is open to question [26]. However, our patients resulted to have viral oral warts, certainly favoured by their immunological status.

Actinic cheilitis resulted significantly more common among adolescents with systemic diseases, especially asthma, diabetes and immunodeficiencies. This is a hardly explainable finding as prolonged exposure to sunlight is the major etiologic factor of actinic cheilitis [26]. We can only speculate that the behaviour (direct exposition to sun or solar lamps) associated to a fair skin and a local/systemic immunosuppression could precipitate the development of actinic cheilitis.

## Conclusion

In comparison to our previous study on the prevalence of OMLs in children, the prevalence in adolescents reflects the transition to adulthood, given the increase of some mucosal conditions as well as the appearance of habits unfamiliar to children. Unlike adulthood, however, preventive interventions in this age group can be very effective.

The majority of lesions and their causes are largely avoidable and could be contained with initiatives promoting oral health, targeted specifically at teenagers. Therefore, public health interventions should aim at reducing various risk factors; from the elimination of local irritants and excellence in dental care, to campaigns designed to prevent compulsive habits and psychological dependencies like smoking and the consumption of alcohol.
